# Binge Drug Injection in a Cohort of People Who Inject Drugs in Montreal: Characterizing the Substances and Social Contexts Involved

**DOI:** 10.1007/s11469-023-01207-7

**Published:** 2023-12-04

**Authors:** Nanor Minoyan, Stine Bordier Høj, Didier Jutras-Aswad, Sarah Larney, Valérie Martel-Laferrière, Marie-Pierre Sylvestre, Julie Bruneau

**Affiliations:** 1 Université de Montréal Hospital Research Centre (CRCHUM), 900 Rue Saint Denis, Montréal, Québec H2X 0A9 Canada; 2 Department of Social and Preventive Medicine, École de Santé Publique, Université de Montréal, 7101 Ave Parc, Montréal, Québec H3N 1X9 Canada; 3 Department of Psychiatry and Addictology, Faculty of Medicine, Université de Montreal, 2900 Boul. Édouard-Montpetit, Montréal, Québec H3T 1J4 Canada; 4 Department of Family and Emergency Medicine, Faculty of Medicine, Université de Montréal, 2900 Boul. Édouard-Montpetit, Montréal, Québec H3T 1J4 Canada; 5 National Drug and Alcohol Research Centre, University of NSW, Anzac Parade, Kensington, 2052 Australia; 6 Department of Microbiology, Infectiology and Immunology, Faculty of Medicine, Université de Montréal, 2900 Boul. Édouard-Montpetit, Montréal, Québec H3T 1J4 Canada

**Keywords:** Binge, Stimulant, Cocaine, Opioid, Overdose, Harm reduction, PWID, Injection drug use, Addiction, Illicit substance use, Prevention, Cohort

## Abstract

We describe binge drug injection in a longitudinal cohort study of people who inject drugs (PWID) in Montreal, Canada (eligibility: age ≥ 18, past-6-month injection drug use; follow-up: 3-monthly interviews). Bingeing was defined as injecting large quantities of drugs over a limited period, until participants ran out or were unable to continue, in the past 3 months. We recorded substances and circumstances typically involved in binge episodes. Eight hundred five participants (82% male, median age 41) provided 8158 observations (2011–2020). Thirty-six per cent reported bingeing throughout follow-up. Binges involved a diverse range of substances and social contexts. Cocaine was involved in a majority of recent binges (73% of visits). Injection of multiple drug classes (24% of visits) and use of non-injection drugs (63% of visits) were common, as were opioid injection (42%) and injecting alone (41%). Binge drug use may thus be an important yet overlooked trigger of overdose and other harms among PWID. This understudied high-risk behavior warrants further research and public health attention.

**Supplementary Information:**

The online version contains supplementary material available at 10.1007/s11469-023-01207-7.

People who inject drugs (PWID) experience a global mortality rate nearly 15 times that of the general population, with leading causes of death relating to overdose and long-term complications of sexually transmissible and blood-borne viral infections (Mathers et al., 2013). These harms persist in many urban settings despite the longstanding presence of harm reduction and high coverage of preventive interventions (e.g. needle-syringe programs and opioid agonist treatment). In such contexts, a large portion of the disease burden associated with injection drug use may be conferred during occasional high-risk consumption episodes.

Binge drug use is generally understood to involve compulsive, high-frequency consumption of substances within relatively limited periods of time. Bingeing may be accompanied by risk behaviours given that individuals’ psychological and physiological state becomes increasingly compromised while engaging in the practice (Bourgois & Bruneau, 2000; Ciccarone, 2011; Tyndall et al., 2003). Although no standardized definition exists, studies using various indicators have previously linked binge-type practices to needle-syringe sharing (Rezaei et al., 2020), non-fatal overdose (Kerr et al., 2007), psychological distress (Minoyan et al., 2021), suicide attempts (Fournier et al., 2018) and HIV infection (Miller et al., 2006) among PWID. However, no studies to our knowledge have reported detailed data on the substances and circumstances involved in binge drug injection. We therefore lack basic evidence to understand the behaviour and address its potential consequences.

For example, exacerbated harms of stimulant (e.g. cocaine, methamphetamine) use are sometimes attributed to binge-type patterns of use in the scientific literature (e.g. Roy et al., 2017a, 2017b). Yet specific mentions or assessments of binge are largely lacking from studies reporting on stimulant use and related harms (e.g. Farrell et al., 2019; Marshall et al., 2011; McKetin et al., 2019). It thus remains unclear whether bingeing is universal among people who inject stimulants, and conversely, whether people exhibit binge-like behaviours when injecting other substances. Specifically, there is a lack of literature characterizing binges of opioids or sedatives, despite their potential role as novel targets for overdose prevention.

Studies in distinct populations (e.g. men who have sex with men; college students) have characterized non-injection binges (e.g. of methamphetamine; alcohol) occurring in the context of social gatherings (Cheng et al., 2010; Guo et al., 2015; Semple et al., 2003). While bingeing with others may facilitate blood-borne virus transmission, this practice also presents an opportunity for bystanders to administer overdose reversal drugs, which can be life-saving. Little is known, however, about the social contexts of binge among PWID.

Considering this dearth of evidence, we sought to describe binge drug injection and the substances involved among PWID in Montreal. We further sought to examine whether PWID commonly binge alone, in addition to characterizing relationships to the people present if and when they binge around others.

## Methods

### Study Population

Data were drawn from a previously described (Minoyan et al., 2020) community-based cohort study of PWID in Montreal, Quebec, Canada (HEPCO). HEPCO was established in 2004 to examine determinants of hepatitis C virus (HCV) infection in this population. Participants are recruited through a combination of approaches including street-level strategies and poster/flyer distribution in community organizations. Individuals are eligible for the study if they are aged 18 years or older, self-report illicit drug injection in the past 6 months and are residents of the greater Montreal area. Since March 2011, HEPCO has followed PWID who have either never been infected with HCV (antibody-negative) or have previously cleared the virus (antibody-positive/RNA-negative). At each 3-monthly visit, trained study staff collect detailed sociodemographic, drug use, behavioural, life event and health service utilization data using a structured questionnaire and administer HIV/HCV testing and pre- and post-test counselling where relevant. Participants who become chronically HCV-infected are followed yearly. Ethical approval was obtained through the institutional review board of the Centre de recherche du Centre hospitalier de l’Université de Montréal (subject to annual renewal). Participants provide written informed consent at enrolment and receive a small monetary compensation for their time.

### Variables

Bingeing was assessed at each visit by asking participants whether, in the past 3 months, they had injected large quantities of drugs, without stopping, over a limited period of time, until they no longer had drugs or were no longer physically able to continue consuming them. The study question was derived from previous research among people who use methamphetamines (Cheng et al., 2010) and refined through consultation with a local group of people who use cocaine, who were asked to name and qualify episodes of intense injection. The resulting definition of a “binge episode” deliberately avoided pre-specifying a duration of use or a quantity of drugs injected, given variations in the psychoactive effects of substances and individual tolerance levels.

Participants who reported bingeing in the last 3 months were asked to estimate how frequently they binged during that referent 3-month period (e.g. weekly, monthly), as well as a series of follow-up questions to characterize the episodes in general terms. These questions aimed to capture what typically occurred during recent binges, rather than alluding to any one specific episode. Participants were asked to report (i) the principal substance(s) injected during recent binges, (ii) the average number of injections per episode, (iii) whether non-injection use of substances occurred (and to name those substances where applicable), (iv) the average duration of episodes, (v) whether binge episodes were generally associated with a loss of control over consumption or long periods without sleep, (vi) whether they shared any needle-syringes during any of these episodes and (vii) whether they always knew where their needle-syringes were. To characterize the social contexts of binge drug injection, we asked participants whether other people were present during any of the binge episodes taking place in the referent period. If no, participants were considered as having exclusively binged alone in the past 3 months. If yes, we asked them to specify their relationships (e.g. friend, acquaintance) to (i) all persons present and (ii) to the person present ‘most of the time’ within that period.

### Analysis

Prevalence of binge and participants’ sociodemographic and drug use characteristics were computed using the first study visit as of March 2011 (the ‘baseline’). To describe binge drug injection, the full repeated-measures HEPCO dataset was restricted to the subset of visits during which participants reported bingeing in the last 3 months. In subsequent sections, we will use the term “binge visits” to convey that the denominator of analyses is the number of study visits with bingeing reported (rather than the number of unique participants or the number of binge episodes). Social contexts of binge were described by gender in this same subset, assessed by asking participants at baseline to specify whether they identified as male, female, or other (no participants replied the latter).

## Results

Between March 2011 and January 2020, 805 individuals (661 men, 144 women, 90% White, 4% Indigenous) contributed 8158 visits to the HEPCO study (median number of visits: 7; Q1–Q3: 3–15). At baseline, the median age was 40.5 [Q1–Q3: 31.6–48.2], and 41.8% of participants were unstably housed or homeless. Two-thirds (66.1%) of the participants reported past-3-month stimulant injection; nearly all of these (92.5%) reported cocaine injection, 30.3% reported amphetamine injection and 22.7% reported injection of both substances. Additional participant characteristics are reported in Table 1. Prevalence of bingeing was 13.5% at baseline, but approximately one-third (35.8%) of the participants reported bingeing at some point over the course of follow-up, for a total of 590 binge visits.

**Table 1:** Characteristics of HEPCO participants at baseline (*N* = 805)

	All (*N* = 805)	
	*n*	%
Age (median [Q1-Q3])	40.5 [31.6–48.2]	
Male gender	661	82.1
Recent incarceration^a^	154	19.1
Housing type^b,e^ *Stable* *Unstable* *Street/shelter*	468 118 218	58.2 14.7 27.1
Indigenous^e^	32	4.0
White^e^	723	90.1
Cocaine injection^c^	492	61.1
Amphetamine injection^c,d^	161	20.0
Any stimulant injection^c^	532	66.1
Heroin injection^c^	294	36.5
Prescription opioid injection^c^	319	39.6
Opioid agonist treatment^a,e^	271	33.7

Assessed in reference to

^a^The past 6 months

^b^The past month

^c^The past 3 months

^d^The study question asked about injection of “speed” prior to 2017

^e^% calculation excludes missing values (maximum *n* = 3)

Almost two-thirds (62.1%) of binge visits featured binge episodes occurring at least once a month. A loss of control when bingeing was reported at 78.5% of the visits, while 29.6% and 48.5% sometimes or always involved long stretches without sleep, respectively. On average, binge episodes lasted 48 h (median value; Q1–Q3: 24–72 h). At 15.5% of visits, the participant reported not always knowing where their syringes were. At 10.6% of visits, the participant reported using a syringe previously used by someone else, and at 6.0%, the participant could not recall whether they had done so.

At a majority (73.2%) of binge visits, the participant reported cocaine injection during recent binges. The next most commonly reported substances were prescription opioids (26.1% of binge visits), heroin (21.2%) and amphetamines (9.0%). Most binge visits (75.9%) involved a single principally injected substance, but 22.2% of binge visits involved both opioids and stimulants (see Table 2). A fifth (20.2%) of binge visits involved only opioids (i.e. heroin or prescription opioids). Concurrent use of non-injection drugs was common (see Table 2): alcohol, snorted/smoked cocaine and cannabis were each reported at roughly one-quarter of binge visits. Overall, the “average” binge episode involved a median of 20 [Q1–Q3: 10–40] injections, though this varied according to substance(s) consumed, and combinations thereof (Table 2).

**Table 2:** Substances involved in binge drug injection in the HEPCO cohort (*N* = 590 visits)

	*n*	%	Average number of injections, median [Q1–Q3]^a^
Principal injected substance(s)^b^:			
Cocaine only Prescription opioid only Heroin only Amphetamine only Cocaine and prescription opioid Cocaine and heroin Heroin and prescription opioid Cocaine and amphetamine Other combination of opioid(s) and stimulant(s)	313 65 47 21 45 43 7 4 43	53.1 11.0 8.0 3.6 7.6 7.3 1.2 0.7 7.1	20 [12–40] 12 [8–30] 8 [4–15] 20 [6.75–48.5] 20 [10–50] 25 [15–40] 16 [10–25] 15 [10–83.75] 20 [12–32.5]
Additional non-injection substance(s)^c^:			
At least 1 non-injection drug Alcohol Cannabis Cocaine/crack (smoked or snorted) Amphetamine^d^ Sedative/tranquilizer Other^e^	371 165 163 142 37 32 24	63.1 28.1 27.7 24.1 6.3 5.4 5.1	N/A

^a^Participants were asked to estimate the average number of injections involved in a typical binge episode occurring in the referent 3-month period; the reported numbers are medians [Q1–Q3] of reported averages

^b^1 missing value excluded from % calculation

^c^2 missing values excluded from % calculation

^d^Question asked about the use of “speed” prior to 2017; the question was subsequently revised to encompass additional types of amphetamine

^e^Computed in the data collected prior to 2017; due to a data input error the variable was missing from the dataset

Regarding social context, the proportion of binge visits at which the participant reported exclusively bingeing alone was 44.0% among men and 29.2% among women. The person present ‘most of the time,’ where applicable, also differed by gender (Table 3). When asked to describe relationships with all persons who were present during binge episodes (i.e. not just the person present most often), response distributions were similar between the two genders; except for romantic partners, who were present during 8.9% of binge visits among men versus 36.0% of binge visits among women (supplementary table I).

**Table 3:** Gender-specific social contexts of binge drug injection (past three months)

	Men (*N* = 477)		Women (*N* = 113)	
	*n*	%	*n*	%
Exclusively binged alone^a^	210	44.0	33	29.2
Binged most commonly with^a,b^:				
*Romantic or sexual partner* *Close friend or family* *Other friend/acquaintance* *Consumption partner* *Stranger* *Other*	46 81 77 45 3 7	9.8 17.3 16.4 9.6 0.6 1.5	33 22 13 8 0 2	29.7 19.8 11.7 7.2 0.0 1.8

^a^Persons present did not necessarily need to be injecting or bingeing during the participants’ binge injection episode(s). Percentages calculated among the subset of visits where participants reported a past-three-month binge injection episode

^b^Denominators used to compute percentages exclude 10 missing values (*n* = 8 among men, *n* = 2 among women)

## Discussion

This study among PWID in Montreal is, to our knowledge, the first to describe substances and circumstances involved in binge drug injection. This work is a first step to better characterizing and subsequently intervening on binge behaviours, which are not a current target of public health messaging and research in this group. Though descriptive in nature, findings are novel and have the potential to inform harm reduction strategies.

Our binge definition aimed to capture drug injection characterized by intense, compulsive use within a restricted period of time. At baseline, 14% of participants reported bingeing in the past 3 months and roughly one-third reported doing so at some point over the course of follow-up. Binge episodes tended to involve a large number of injections, loss of control over consumption and long sleepless periods; these factors may in turn contribute to impaired decision-making and lapses in preventive behaviours, consistent with studies linking binge to sharing of injection equipment (Rezaei et al., 2020; Wood et al., 2002) and HIV transmission (Miller et al., 2006). Binges may also exacerbate risk of overdose (Kerr et al., 2007) and psychiatric and psychosocial harms (Cheng et al., 2010; Lappin & Sara, 2019; McKetin et al., 2019) including increased psychological distress (Minoyan et al., 2021) and suicidality (Fournier et al., 2018), as documented among PWID.

Binge is sometimes conflated with stimulant use due to the consumption patterns that stem from the short half-life of this drug class (e.g. Substance Abuse & Mental Health Services Administration, 2021; Tyndall et al., 2003; World Health Organization. Regional Office for the Western Pacific., 2011). Consistent with these descriptions, binge injection in our cohort tended to involve one or more stimulant (primarily cocaine). Interestingly, however, bingeing was not ubiquitous among those who injected stimulants: at baseline, only 18% of cohort participants reporting past-3-month stimulant injection reported bingeing in that period (data not shown). These occasional binges may nevertheless disproportionally contribute to stimulant-related harms, highlighting the urgent need for novel pharmacological therapies for stimulant dependence (Ronsley et al., 2020).

Binges did not exclusively involve stimulants, however. An unexpected and novel finding was the substantial proportion of participants reporting exclusive injection of opioids during recent binges (one-fifth of binge visits). Prior research from Montreal suggests that PWID inject prescription opioids numerous times a day to stave off withdrawal symptoms when doses available to them are suboptimal, and also often re-inject with “washes” — rinses of residue left behind by previous injections (Roy et al., 2011). Although such practices may satisfy the definition of bingeing used in our study, and indeed carry important infection- and overdose-related risks, they may not engender the same psychosocial consequences as compulsive, frenetic injection patterns more typical of stimulant bingeing (Bourgois & Bruneau, 2000; Ciccarone & Bourgois, 2016; Roy et al., 2017a, 2017b). Research is thus needed to better understand how people who use opioids versus stimulants perceive and experience binges. Targeting reductions in bingeing may be a fruitful public health endeavour in the former group given the existence of effective opioid agonist treatments.

Many participants reported multiple substance classes when asked to name the principal substances injected during recent binges: almost a quarter of binge visits involved injection of both opioid(s) and stimulant(s) and almost two-thirds involved additional use of non-injection substances. Alcohol, which is itself prone to binge patterns of consumption, was involved in around one-quarter of binge visits. Co-use may potentiate drug-related harms, including risk of overdose (Witkiewitz & Vowles, 2018), as well as liver-related complications among those infected with HCV (Alavi et al., 2018).

A concerning finding was the high proportion reporting that they were usually alone during binges: over half of the individuals who reported bingeing reported that they had binged alone at some point during the follow-up, with men reporting this risk behaviour more often than women. Results of qualitative studies examining motivations for *injecting* alone suggest concerns for personal safety, as well as a desire for privacy while experiencing drug effects (Rosen et al., 2022). While some of these motivations may be similar for *bingeing* alone, this practice may exacerbate the risk of opioid-related death by overdose. Individuals who binge should be encouraged to visit supervised consumption sites, which in turn should be tailored to their needs. This is particularly salient for people who inject stimulants, who may display more erratic behaviours than people injecting opioids, as well as for women who inject drugs, who may feel alienated from harm reduction sites that infrequently take into account their specific vulnerabilities (El-Bassel & Strathdee, 2015).

For those who binge with others, the social contexts identified in this study, combined with the finding that binge episodes commonly involve opioids, have implications for the prevention of fatal overdoses caused by bingeing. Participants overwhelmingly knew the people who were present during binge episodes, and they most often reported close relationships. Equipping all PWID with naloxone is an important public health endeavour regardless of individual-level patterns of drug use and drugs of choice; its availability in a person’s social network can be life-saving in cases where those who binge experience an overdose event.

It remains unclear to what extent binge drug use among PWID occurs in the context of social gatherings or club/party scenes, as described, for instance, in studies focused on amphetamine use (Jenkinson et al., 2014; Kelly et al., 2013). Although bingeing with others may constitute an overdose risk reduction measure among PWID, such gatherings may simultaneously drive higher-intensity injection through peer effects, as described for alcohol use (e.g. Guo et al., 2015) and facilitate blood-borne virus transmission (Bradshaw et al., 2016). It thus remains fundamental to ensure that all PWID are equipped with sterile injection materials, including ancillary equipment such as cookers (Roy et al., 2017a, 2017b). 1:1 syringe exchange models still present in certain jurisdictions should be replaced with distribution-based models to maximize coverage for each individual injection, as well as to maximize population-level impacts (Sherman et al., 2015). Expecting individuals to anticipate a set number of injections for a later binge is unrealistic and antithetical to the compulsive nature of the practice.

A first step towards developing psychosocial interventions would be to better elucidate whether individuals perceive binge drug injection as a modifiable practice or as an inextricable aspect of their dependence on certain substances. Understanding the specific contexts or mental health states that favour deliberate engagement in binge behaviour may support the development of public health responses. Qualitative or event-level analyses, which aim to characterize individual instances of binge, may provide particular insight into motivations for bingeing and psychosocial triggers of this behaviour.

### Limitations

There is no standardized definition of a binge episode. Studies have operationalized this behaviour in various ways ranging from definitions analogous to ours (Cheng et al., 2010; Semple et al., 2003) to definitions referring to use of drugs at a ‘greater-than-usual’ frequency or intensity (e.g. Bozinoff et al., 2017; Scott et al., 2016), or referring to a duration of use without sleep (Bruno et al., 2009). Our indicator, which was adapted for stimulant-using populations in collaboration with people with lived experience, may under-capture binge injection phenomena among people who inject opioids or other substances.

Second, participants described what bingeing typically “looked like” in the last 3 months. It follows that the 22% reporting multiple substances as their principally injected drugs may not have been injecting those substances within the same binge episodes.

Third, many binges occurring in this population might not be injection binges but rather smoked crack or alcohol binges, which were not captured in this study. Binge behaviour as a general high-risk practice would therefore be underestimated in our cohort. While these modes of consumption may elicit distinct harms and require further consideration, we believe that our precise definition captures an understudied phenomenon with important relevance for blood-borne virus and overdose prevention.

Fourth, there is potential for bias due to imperfect recall of binge episodes and substances used. Participants may also have omitted reporting about certain practices due to social desirability bias. However, study interviewers were trained and experienced in interacting with the study population and used the timeline follow-back method (Fals-Stewart et al., 2000) to elicit answers. Furthermore, similar studies have reported high agreement between self-reported and biological measures of illicit drug use (Bharat et al., 2023).

Finally, differential loss-to-follow-up may have contributed to an over-representation of certain participants. However, we believed that the large number of binge visits available in the HEPCO dataset warranted using the full set of repeated measures to address the aims of the investigation. Additionally, participants provided a median of 1 [Q1–Q2: 1–2] observations to the binge subset, and those lost to follow-up after one visit (*n* = 132) did not substantially differ from those retained (*n* = 673) in terms of binge prevalence at baseline (15.2% vs. 13.2%, respectively).

## Conclusion

Binge drug injection may be an under-considered driver of harms among PWID and may constitute a novel behavioural target in this population. Its close association with stimulant use reinforces the dire need for suitable and effective pharmacological therapies. Future work should seek to characterize the role of opioids and non-injection drugs within binges and the potential impacts of interventions such as naloxone and needle-syringe distribution specifically targeting binge episodes.

## Supplementary Information

Below is the link to the electronic supplementary material.

Supplementary file1 (DOCX 17 KB)

## Data Availability

The data that support the findings are available on request due to confidentiality/ethics restrictions. Data are not publicly available as individual-level information may identify participants.

## Code Availability

Code for statistical analysis available upon request.
