# Supplementary material for: Binge Drug Injection in a Cohort of People Who Inject Drugs in Montreal: Characterizing the Substances and Social Contexts Involved
Source: Int J Ment Health Addict. 2023 Dec 4;23(3):1908–19. doi: 10.1007/s11469-023-01207-7 (PMC12279887; doi:10.1007/s11469-023-01207-7)
Supplement: Supplementary file 1 — Supplementary file1 (DOCX 17 KB) [file 11469_2023_1207_MOESM1_ESM.docx]

**Supplementary table I: Gender-specific social contexts of binge drug injection (past three months)**

|  | **Men (N=477)** | | **Women (N=113)** | |
| --- | --- | --- | --- | --- |
|  | **n** | **%** | **n** | **%** |
| **Exclusively binged alone^a^** | 210 | 44.0 | 33 | 29.2 |
| **Binged with^a,b^:**  *Romantic or sexual partner*  *Close friend or family*  *Other friend/acquaintance*  *Consumption partner*  *Work-related acquaintance^c^*  *Stranger*  *Other* | 52  98  107  67  8  14  1 | 10.9  20.6  22.5  14.1  1.7  2.9  0.2 | 44  33  32  18  6  2  2 | 38.9  29.2  28.3  15.9  5.3  1.8  1.8 |

1. Persons present did not necessarily need to be injecting or bingeing during the participants’ binge injection episode(s). Percentages were calculated among the subset of visits at which participants reported past-three-month binge injection.
2. Participants were asked to report relationships to any persons present during past-three-month binge injection episodes. Denominators used to compute percentages exclude 2 missing values among men.
3. Any work-related acquaintance not applicable to the other categories (e.g. client, pimp, dealer) was classified in this group.
